# Supplementary material for: Ethanol as a Probe for the Mechanism of Bubble Nucleation in the Diet Coke and Mentos Experiment
Source: Molecules. 2021 Mar 17;26(6):1691. doi: 10.3390/molecules26061691 (PMC8002754; doi:10.3390/molecules26061691)

1. A video that outlines how to build and use foam volume device can be found here: <https://www.youtube.com/watch?v=NNaLjQEUIrs>
2. Pictures of the assembly used to measure time-dependent foam volume is seen below. For consistent delivery, a Mentos candy is dropped through the tube (pictured on the left) and into the soda. The tube is immediately removed so as not to interfere with the foam production (picture on the right).


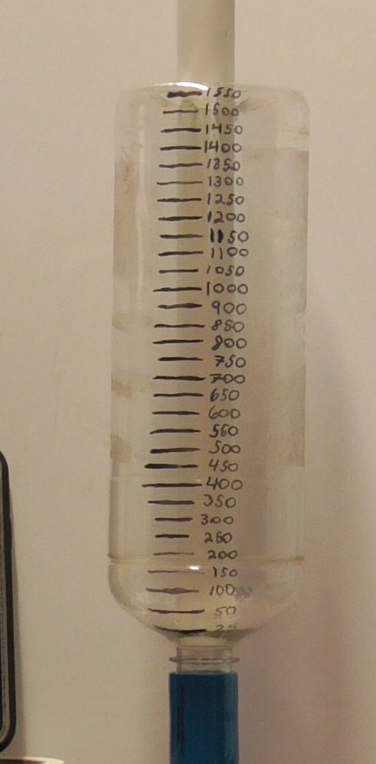

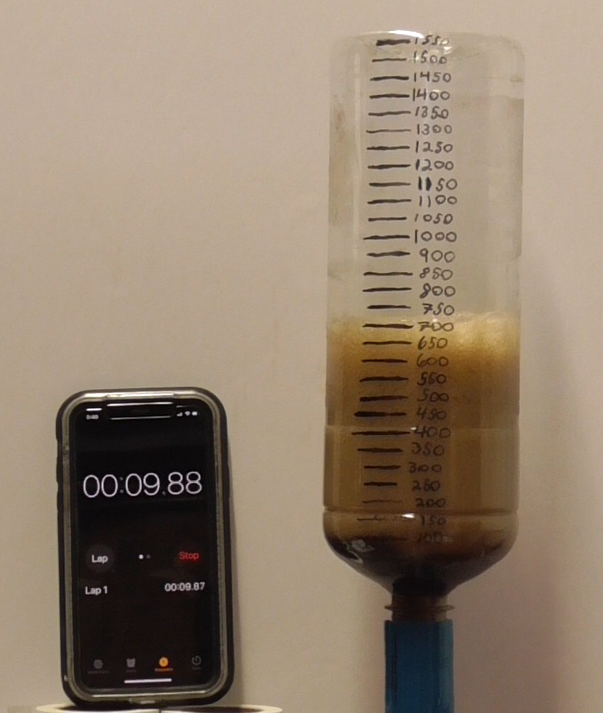


1. A frame of video captured of the fountain induced when 11 Mentos candies were dropped into a prepared 2L sample of Diet Coke is displayed below. The video was paused at the moment the fountain reached its maximum height. The actual distance from the ground level to the bottom of the overhang (represented by the red line) was measured to be 2.60 m. The fountain height from the top level of the liquid (prior to adding Mentos) to the maximum foam height is represented by the yellow line. On the video play back screen, the red line was measured to be 8.85 cm, and the yellow line was measured to be 5.90 cm, yielding a measured actual fountain height of 1.73 m.


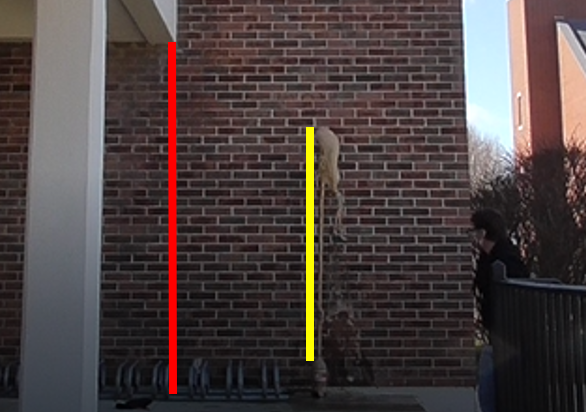

Supplement: Supplementary file 1 [file molecules-26-01691-s001.zip › Supplementary information/Picture and video link for foam volume device.docx]
